# Extension disorders of fingers with congenital anomalies of the flexor digitorum profundus: a case report

**DOI:** 10.3389/fsurg.2025.1670938

**Published:** 2025-11-06

**Authors:** Benbiao Wang, Deguo Luo, Naiqiang Zhuo, Yulin Xu, Jianhua Ge

**Affiliations:** 1 Department of Orthopedics, The Affiliated Hospital, Southwest Medical University, Luzhou, Sichuan, China; 2 Sichuan Provincial Laboratory of Orthopaedic Engineering, Luzhou, Sichuan, China

**Keywords:** congenital, flexor digitorum profundus, flexion deformity, aberrant origin, muscle sliding

## Abstract

**Introduction:**

An aberrant origin of the deep flexor muscle causing congenital flexion deformity of the middle, ring, and little fingers is relatively atypical, and its etiology remains unclear. Previous reports indicate that surgeons have employed a muscle-sliding procedure of the flexor muscles to treat the condition. However, postoperative loss of motion was observed. In our patient, we enhanced the original surgical method by incorporating intramuscular extension of the superficial digital flexor muscle, along with close follow-up and guidance on functional exercises, leading to improved outcomes for the patient. These results may serve as a valuable reference for clinical practice.

**Main symptoms and important clinical findings:**

The physical examination demonstrated the three affected fingers showed flexion deformity upon wrist extension. The three fingers could be actively extended when the wrist was flexed. During the examination, the patient experienced no pain, and the thumb and index fingers exhibited normal motion. The diagnostic assessment was completed with x-ray, 3D CT, and MRI of the forearm.

**Therapeutic interventions and outcomes:**

The patient underwent surgery: the bony process was excised, followed by severing of the tendinous band, the contracted tissues at the deep flexor tendon origins were further released toward the radial side. Muscle sliding was followed by the resection of the hyperintense tendinous portion of the superficial flexor. Postoperatively, the wrist joint was externally fixed using plaster at 30° dorsiflexion, and the fingers were extended for 3 weeks, along with close follow-up and guidance on functional exercises, Finally, the affected side is functionally equivalent to the unaffected side.

**Conclusion:**

The incidence of this condition is relatively low, but diagnosis is relatively straightforward. It requires differentiation from other finger deformities. Based on the degree of contracture, the patient's age, and findings during the intraoperative examination, an appropriate surgical approach should be selected. Combined with close follow-up and functional exercise guidance, favorable treatment outcomes can be achieved.

## Introduction

A significant challenge for orthopedists is the reconstruction of the hand function. Flexor tendon contracture of multiple fingers typically occurs because of scar adhesion or muscle degeneration following trauma. Volkmann's contracture, a muscle movement disorder characterized by irreversible fibrosis and muscle fiber necrosis, is a common cause (1). Congenital multiple finger flexor contracture, a rare disorder, has multiple etiologies, including Dupuytren's disease. This condition is characterized by scar tissue growth in the palm and is surgically managed by excising the affected fascia tissue and releasing the tendon. The diagnosis is confirmed through postoperative histological and pathological examination (2).

In 2008 (3) and 2022 (4), Xiong et al. reported a unique case of flexion contracture affecting the middle, ring, and little fingers in the neutral position of the wrist joint. The three fingers in this patient could achieve extension when the wrist joint was flexed. The presence of an abnormal bony process and a binding band structure was confirmed at the ulnar origin of the deep flexor tendon affecting the three fingers using both three-dimensional (3D) computed tomography (CT) imaging and intraoperative findings. A muscle-sliding procedure was performed on the flexor muscles, thus permitting the wrist to achieve 60° of dorsal extension and enabling passive finger extension. However, loss of motion was observed postoperatively. Moreover, the simple muscle-sliding procedure was deemed inadequate for restoring the full range of motion in the affected hand. Herein, we present a relatively successful case of this condition.

## Case presentation

On July 5th, 2022, a 15-year-old boy presented with the chief complaint of impaired extension in the left middle, ring, and little fingers and was subsequently admitted. The patient had not exhibited any symptoms of finger flexion contracture before the age of 7 years. He had no previous history of trauma, intrapartum injuries, or familial finger contractures.

**Figure 1:**
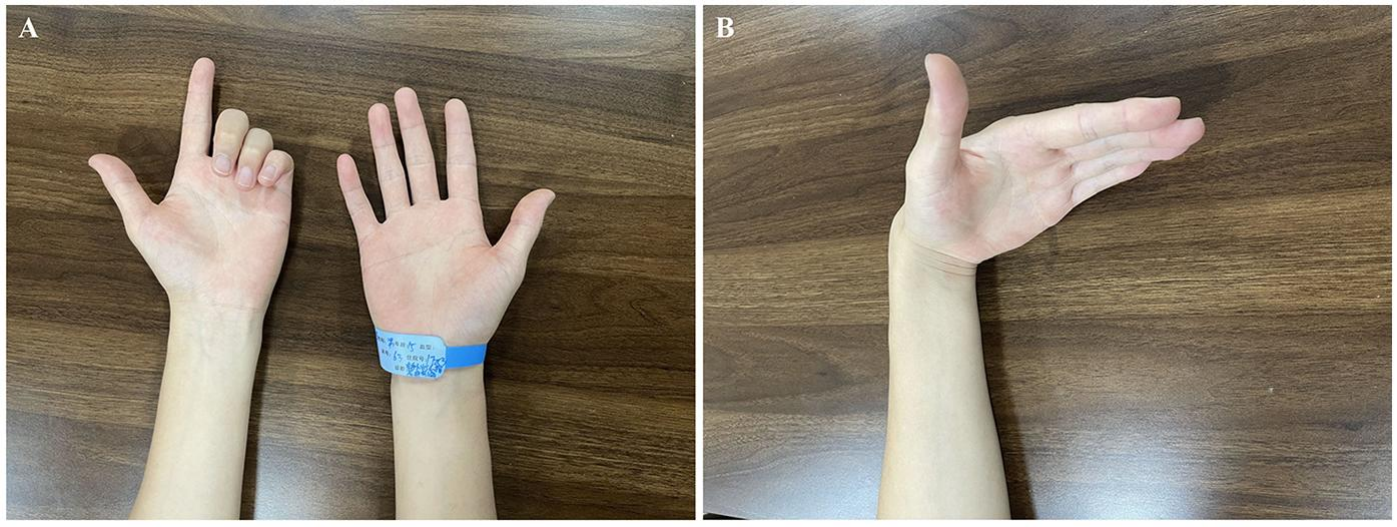
Hand appearance: **(A)** when the wrist joint is extended, the middle, ring, and little fingers exhibit flexion deformity and impaired active extension. **(B)** When the wrist joint is in a flexed position, the middle, ring, and little fingers can be actively extended.

The physical examination demonstrated a normal appearance of the left forearm and hand, with normal skin sensation and no scar nodules. The three affected fingers showed flexion deformity upon wrist extension. The patient exhibited impairment and significant resistance during active and passive extension, respectively. The three fingers could be actively extended when the wrist was flexed (Figure 1). During the examination, the patient experienced no pain, and the thumb and index fingers exhibited normal motion.

### Diagnostic assessment

**Figure 2:**
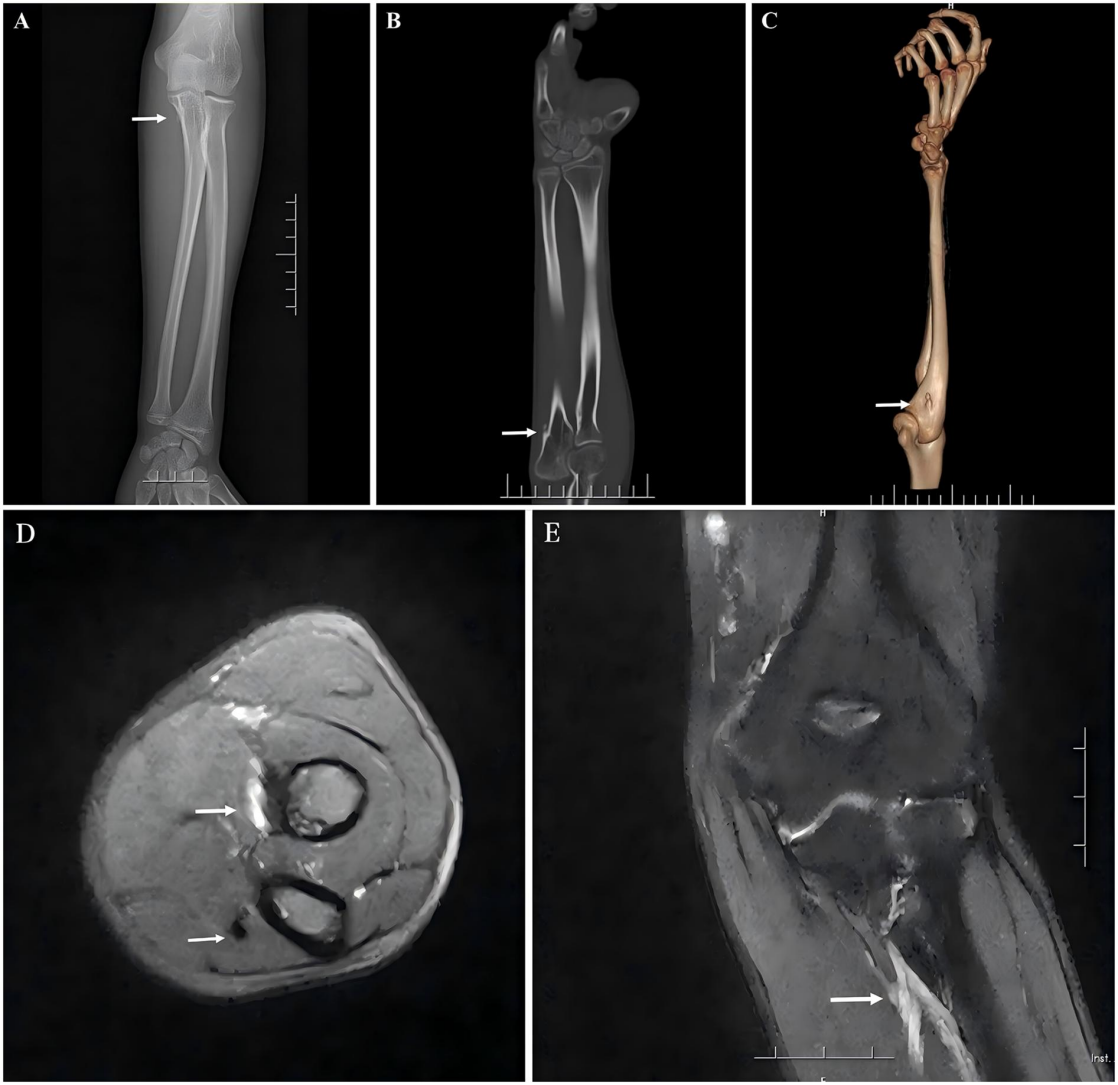
**(A,C)** x-ray and 3D CT scans reveal a bony prominence on the medial aspect of the proximal ulna (indicated by arrow). **(B)** The coronal CT scan of the ulna shows that the bony prominence originates from the cortical surface of the ulna, extending distally (indicated by arrow). **(D)** MRI shows a high-intensity lesion in the deep muscle layer of the anterior proximal forearm and an abnormal fibrous cord in the flexor digitorum profundus. **(E)** A high-intensity lesion in the deep muscle layer of the anterior proximal forearm.

The anteroposterior x-ray, 3D CT, and MRI of the forearm (Figure 2) show that a small, approximately 0.4 cm long nodular bony process was revealed on the proximomedial aspect of the left ulna, with a broad base extending dorsally toward the articular surface. A hyperintense lesion was revealed in the deep layer of the proximal forearm muscles through T2-weighted magnetic resonance imaging of the forearm, along with thickened fiber bundles at the ulnar attachment of the flexor digitorum profundus. Based on his symptoms and auxiliary examinations, we reached a diagnosis: congenital progressive contracture of the flexor digitorum profundus in the forearm.

### Therapeutic intervention

**Figure 3:**
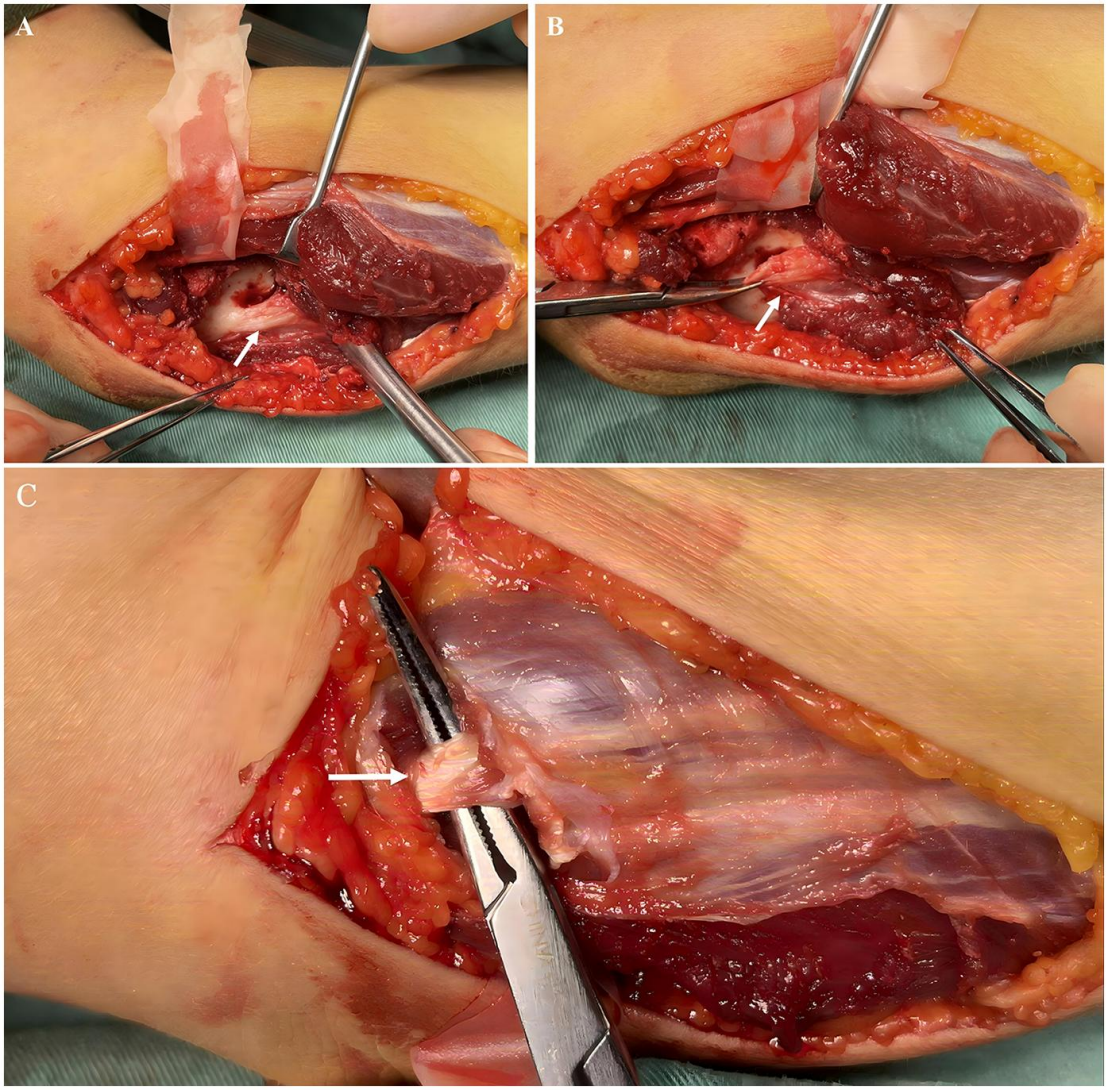
**(A)** The Anomalous bone prominence (indicated by arrow). **(B)** The anomalous tendinous band attached to the bone prominence (indicated by arrow). **(C)**The superficial flexor tendon is cut (indicated by arrow) while preserving the surrounding muscles and fascia.

The patient has experienced progressively worsening finger extension difficulties over the course of nearly eight years. After a period of ineffective conservative treatment, the patient and his family opted for open surgery. An incision was made on the proximomedial surface of the forearm extending to the deep fascia, and the ulnar nerve and its first muscular branch were exposed between the deep and superficial flexor muscles. The deeper tissues were exposed by cautiously extending the incision. The distally growing bony process was identified and connected to the flexor digitorum profundus tendon attachment of the three affected fingers using an abnormally thickened tendinous band (Figures 3A,B). The bony process was excised, followed by severing of the tendinous band. The flexion deformity of the three fingers improved significantly, with the fingers becoming straight in the neutral wrist position. However, the fingers did not remain straight when the wrist was flexed. Consequently, the contracted tissues at the deep flexor tendon origins were further released toward the radial side. Muscle sliding was followed by the resection of the hyperintense tendinous portion of the superficial flexor. Intramuscular lengthening was subsequently performed (Figure 3C) with meticulous protection of the aponeurosis. The examination of the hand was performed to confirm that the three fingers could be completely extended passively when the wrist was extended to 60°. Postoperatively, the wrist joint was externally fixed using plaster at 30° dorsiflexion, and the fingers were extended for 3 weeks. Pathological examination of the intraoperatively excised bony process and hyperplasia demonstrated mature bone and fibrous connective tissues with hyperplastic hyaline. Passive functional exercises were gradually initiated starting 1 day postoperatively, with the assistance of the rehabilitation clinicians. The patient was instructed to perform exercises in the morning and afternoon (30 min each) using a step-training style, with the wrist joint progressing through 0°, 10°, 20°, and so on. At 70° of wrist flexion, the fingers were passively extended to the extreme dorsiflexion position and held for 5 s per cycle. The cycles were repeated until the pain was tolerable for the patient.

### Follow-up and outcomes

**Figure 4:**
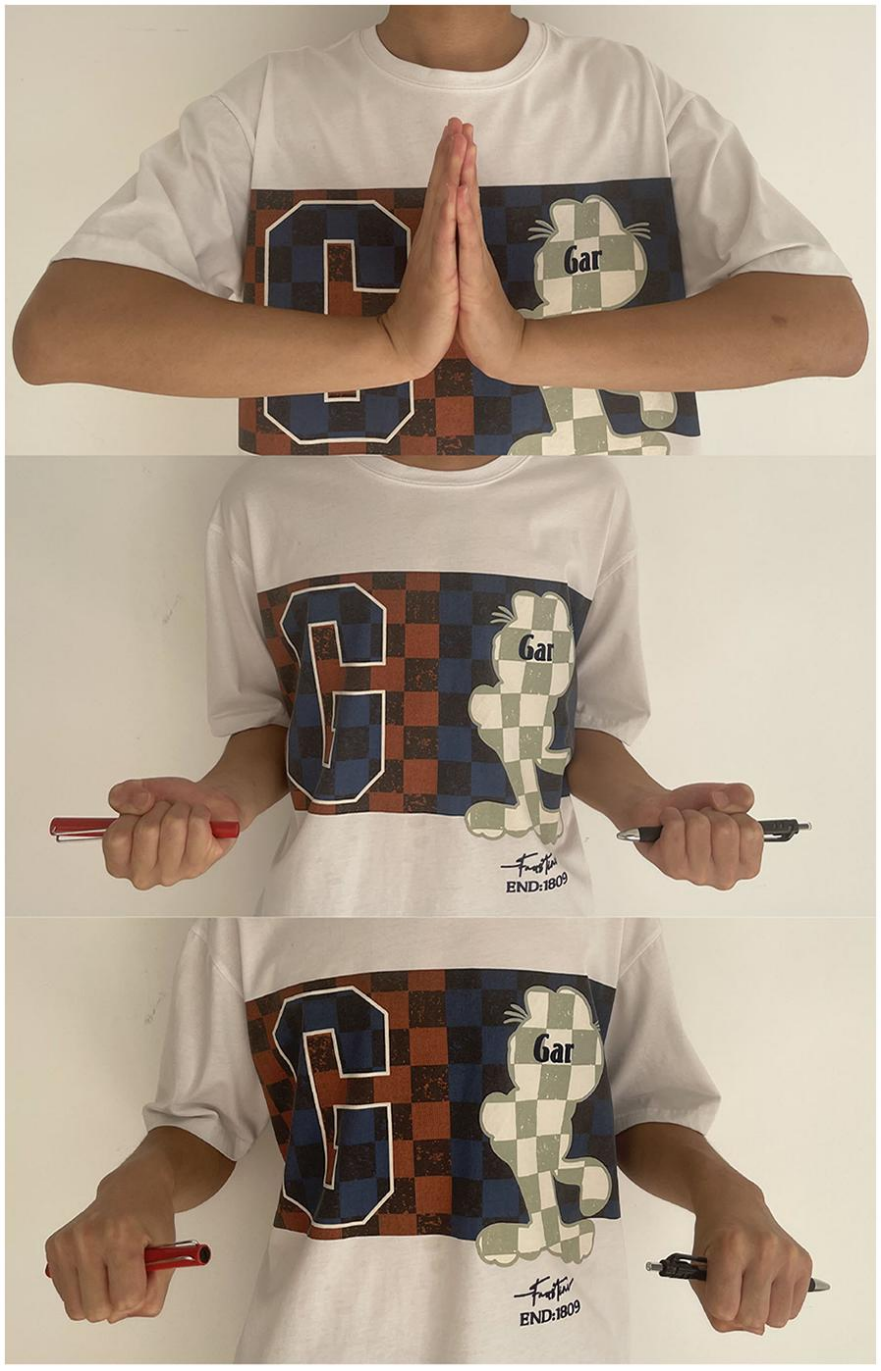
Upper limbs mobility 13 months after the performance of the surgery.

The patient was followed up at 1, 2, 3, 6, 12 weeks, and 13 months post-discharge. The function of the affected side was comparable to that of the unaffected side by week 12. The left-to-right hand grip ratio was 0.79. In his last clinical examination, 13 months after the surgery, the little finger and the middle and ring fingers could be extended when the wrist joint was extended to its extreme position, and the forearm rotation was normal (Figure 4). Finally, the patient could resume his daily and sporting activities.

## Discussion

Although similar previous literature exists, the original surgical treatment was modified by adding an intramuscular extension of the superficial digital flexor muscle and combining it with functional exercise guidance. Consequently, improved clinical outcomes were observed in the patient. Both the previously reported and present cases exhibited abnormalities in the bony process and origin of the flexor digitorum profundus. The bony processes were speculated to have formed early, subsequently affecting the normal development and growth of the flexor digitorum profundus. The abnormalities of the tendon progressively restricted finger extension. However, the cause of the bone process formation could not be confirmed through genetic evidence. Moreover, postoperative histopathological findings could not elucidate this condition.

In young patients with muscle contracture who have no muscle necrosis or nerve injury, the muscle sliding procedure is suitable. Muscle gliding techniques adhere to the principle of muscle resting length, maintaining the proportional relationship between muscle and tendon length units. This approach preserves strength more effectively than Z-plasty when lengthening short tendons. The muscle sliding procedure is widely used in plastic surgery for moderate to severe Volkmann's contracture deformities, with satisfactory clinical outcomes and low rates of recurrence (5–7). Even when the surgery is unsuccessful, the second stage can be managed using tendon transposition. However, the muscle should be carefully dissected outside the periosteum during the operation to prevent damage to the periosteum, consequently diminishing the risk of postoperative ectopic ossification. The persistent flexion deformity inevitably led to the shortening of the flexor digitorum superficialis because of the prolonged course of the disease in this case. After muscle sliding, the distal finger could not be extended when the wrist joint was dorsiflexed. Consequently, the intramuscular extension of the superficial flexor digitorum was performed to improve function. Intramuscular lengthening facilitates muscle continuity extension while preserving muscle integrity (8), lowers the risk of excessive correction and adhesion, and permits adaptive muscle extension in accordance with the range of motion during functional exercise. The patient achieved good hand function in subsequent follow-up. In younger children, simple resection of the fibrous band can yield good results (9).

The repair and reconstruction of contracture deformities are performed using tendon lengthening. This technique relies on the anatomical structure of the muscle with a contraction function to restore the physiological length of the tendon. The degree of surgical correction should be accurately calculated before the surgery, or else muscle and joint strength could be compromised postoperatively (10). The extension is often excessive to minimize contracture recurrence and maximize limb functional activities, and postoperative protective fixation may increase the risk of adhesion. Takagi et al. (11) reported middle finger flexion deformity in an 8-year-old boy, with symptoms similar to those in our case. Tendon severing and transposition were performed to the fourth finger's deep flexor tendon, obtaining favorable outcomes. This summary presents previously reported cases (Table 1). However, tendon transposition is more commonly recommended in patients with neurological diseases or severe muscle injuries that cannot be repaired and in those with poor outcomes after prolonged release. Therefore, these tendon severing and transposition procedures may not be recommended as the first alternative for such patients.

**Table 1:** Clinical characteristics and treatment of congenital deep finger flexor deformity with finger extension impairment in the literature.

Literature	Age/sex	Clinical symptoms	Treatment measures	Follow-up duration	Outcome
Xiong et al. (3)	21Y (M)	When the wrist joint is in the resting position, the middle, ring, and little fingers are flexed, allowing the fist to be clenched; when the wrist joint is in the neutral position, fingers extension are limited.	Excision of the abnormal origin of FDP and tendon sliding surgery for deep flexor muscles	1 year	After full release, passive extension of the middle, ring, and little fingers was possible with the wrist in neutral position
Xiong et al. (4)	4–30Y (7M, 1F)	When the wrist joint is in the resting position, the middle, ring, and little fingers are flexed, allowing the fist to be clenched; when the wrist joint is in the neutral position, fingers extension are limited. (7 left, 1 right)	Excision of the abnormal origin of FDP and tendon sliding surgery for deep flexor muscles	0.5–12 years	Wrist extension increased by 20–40 degrees in adults; in younger patients, no significant effect on finger extension at wrist 0°; grip strength recovered to 82% of the unaffected side
Li et al. (9)	1–24Y (5M, 5F)	Flexion deformity of the middle, ring, and little fingers at the wrist joint in the neutral position (4 cases), Flexion deformity of the middle and ring fingers (3 cases), Flexion deformity of the ring and little fingers (2 cases), Flexion deformity of a single finger (1 case)	6 cases were performed under local anesthesia; abnormal fibrous bands were severed at the connection between the deep flexor tendon and the proximal ulna	2–26 months (mean 16 months)	8 cases had a good prognosis; 2 cases (youngest age, poor compliance) had a fair prognosis
Takagi et al. (11)	8Y (M)	The proximal interphalangeal joint and distal interphalangeal joint cannot fully extend the middle finger when the wrist is in a neutral position.	The excised middle finger FDP tendon was cross-sutured with the ring finger FDP tendon, and the ring finger fascia was lengthened in segments	1 year	Satisfactory recovery of middle finger function
Zhang et al. (17)	16–20Y (2M, 1F)	Flexion deformity of the proximal and distal interphalangeal joints of the middle, ring, and little fingers in neutral or extended wrist position; deformity worsened with wrist extension	Excision and severing of thickened tendinous tissue; one patient underwent Z-plasty to lengthen the shortened tendon	12, 35, and 12 months	Significant improvement in hand function and joint range of motion, but no significant improvement in grip strength; 2 cases were excellent, 1 case was good

M, male; F, female; FDP, flexor digitorum profundus.

Early detection during childhood and timely diagnosis, which depend on prompt identification in the outpatient department and treatment is expected to reduce surgical trauma and lead to better outcomes. First, the flexion contracture of the middle, ring, and little fingers, owing to the abnormal origin of the flexor digitorum profundus, demonstrates no familial aggregation or history of labor-related injuries or trauma. The onset of the disease is predominantly unilateral. Only one or two fingers on the ulnar side may be affected in some children (9). Additionally, finger flexion contracture occurs in both the neutral and dorsal extension positions of the wrist joint. The fingers can be extended when the wrist joint is in flexion, with normal metacarpophalangeal and interphalangeal joint functions; the intrinsic muscle function of the hand is intact, with no scar nodules in the local skin. In contrast, Dupuytren's disease (12) is a benign fibroproliferative condition where contracture of the palmar aponeurosis develops, causing a rigid flexural deformity of the knuckles. Trismus-pseudocamptodactyly syndrome (13), an autosomal dominant congenital disorder, is clinically characterized by a reduced ability to open the mouth and finger curvature at the level of the interphalangeal joints while attempting dorsiflexion of the wrist (pseudocamptodactyly). Volkmann ischemic contracture (14) is a permanent flexion deformity caused by ischemia, resulting in a claw-like appearance of the hand. The primary triggering event is acute compartment syndrome, typically caused by trauma leading to fractures or soft tissue crush injuries, or vascular complications. CACP Syndrome (15) typically manifests as flexion contractures at the proximal interphalangeal joints, often accompanied by swelling of the large joints but without typical inflammatory signs such as redness, heat, or pain. Some patients with hip dysplasia may develop serous cavity effusions. Ultimately, the gold standard relies on molecular diagnosis of PRG4. Yan et al. (16) through gene sequencing identified a mutation in exon 26 of the Fibrillin 2 gene as the causative factor for congenital finger flexion. However, clinical manifestations of congenital finger flexion vary among different types, and further studies are required to provide more insights.

## Conclusions

Congenital flexion deformities of the middle, ring, and little fingers, accompanied by abnormal origins of the flexor digitorum profundus (FDP) tendon, are rare, and their etiology remains unclear. Through detailed inquiry into the patient's medical history combined with a physical examination, along with x-ray or CT imaging studies, this condition can be identified. The diagnosis is straightforward, and appropriate surgical methods are selected based on the degree of contracture, the patient's age, and findings during the intraoperative examination, along with close follow-up and guidance on functional exercises, thus yielding favorable treatment outcomes.

### Patient perspective

Our case demonstrates that the patient has had a good clinical outcome. 13 months after the surgery, the three affected fingers could be extended when the wrist joint was extended to its extreme position, and the forearm rotation was normal. The patient could resume his daily and sporting activities finally.

## Data Availability

The original contributions presented in the study are included in the article/Supplementary Material, further inquiries can be directed to the corresponding author.
